# Enhanced humoral immunity in breast cancer patients with high serum concentration of anti‐HER2 autoantibody

**DOI:** 10.1002/cam4.3742

**Published:** 2021-01-27

**Authors:** Yasufumi Sato, Masafumi Shimoda, Yoshiaki Sota, Tomohiro Miyake, Tomonori Tanei, Naofumi Kagara, Yasuto Naoi, Seung Jin Kim, Shinzaburo Noguchi, Kenzo Shimazu

**Affiliations:** ^1^ Department of Breast and Endocrine Surgery Osaka University Graduate School of Medicine Suita Osaka Japan; ^2^ Hyogo Prefectural Nishinomiya Hospital Nishinomiya Hyogo Japan

**Keywords:** B‐lymphocytes, CD4‐positive T‐lymphocytes, humoral immunity, tumor microenvironment, tumor‐infiltrating lymphocytes

## Abstract

Humoral immunity plays a substantial role in the suppression of breast cancer. We have revealed that a high serum concentration of anti‐HER2 autoantibody (HER2‐AAb) is associated with favorable outcomes in patients with invasive breast cancer. Thus, we aimed to clarify the association between high serum concentration of HER2‐AAb and humoral immune response in the tumor microenvironment. Out of 500 consecutive patients with invasive breast cancer, we selected those whose HER2‐AAb values were high (*n* = 33) or low (*n* = 20) based on the distribution of HER2‐AAb values of 100 healthy individuals. Tumor and regional lymph node formalin‐fixed paraffin‐embedded samples prepared from the surgical specimens were subjected to immunohistochemistry. We confirmed that the recurrence‐free survival of the high HER2‐AAb group was significantly longer than that of the low HER2‐AAb group (*p* = 0.015). The numbers of tumor‐infiltrating CD20+ immune cells (ICs) (*p* < 0.001), IGKC+ICs (*p* = 0.023), and CXCL13+ ICs (*p* = 0.044) were significantly higher in the high HER2‐AAb group than in the low HER2‐AAb group. The number of CD4+ ICs in the B‐cell follicles of the regional lymph nodes was also significantly greater in the high HER2‐AAb group than in the low HER2‐AAb group (*p* = 0.026). Our findings indicate that a high level of HER2‐AAb is associated with enhanced humoral immunity against breast cancer and thus may provide a rationale for the association of HER2‐AAb with favorable prognosis.

## Graphical abstract

Anti‐HER2 autoantibody in breast cancer patients is positively associated with their outcomes. Enhanced humoral immunity in the tumor microenvironment is associated with autoantibody, providing a rationale for favorable outcomes in such patients.

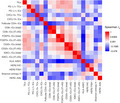

## 1 INTRODUCTION

Studies on autoantibodies found in cancer patients began in the 1950s, and the findings have been rapidly accumulating since the 1980s.^1^, ^2^ Although the mechanism underlying the production of autoantibodies in cancer patients remains largely unknown, several findings have linked autoantibody production with autoimmune response to cancer. Autoantibodies to RPC1, an RNA polymerase subunit encoded by the POLR3A gene, emerge in the sera of scleroderma patients with cancer in whom the POLR3A gene is mutated. Moreover, ex vivo data show that somatic POLR3A mutations in cancer patients trigger an autoimmune response.^3^ Notably, autoantibodies to various proteins can be found in more than half of the patients with breast cancer,^4^ suggesting that breast cancer is one of the cancer types strongly associated with autoantibody production. During breast carcinogenesis, the tumor‐associated antigens (TAAs) of centrosomes, centromeres, and mitochondria trigger an antigen‐driven autoimmune response, leading to the production of autoantibodies to those antigens in patient sera, and such an immune response is distinct from that in the individuals without breast cancer.^5^ Subtype‐specific autoantibody repertoires are also demonstrated in patients with breast cancer; autoantibodies to spliceosomes and glycolysis‐related proteins are observed in luminal breast cancer, while autoantibodies to BRCA1, TP53, and cytokeratin‐5/6/14 are often observed in patients with mesenchymal/basal‐like breast cancer. These findings suggest that autoantibodies are produced in breast cancer patients according to the proteins present in breast cancer cells. Thus, the enhancement of humoral immune response may be a predominant feature of breast cancer.^5^, ^6^, ^7^

Despite the association of breast cancer with autoantibodies, there are very few reports on the association of autoantibodies with breast cancer prognosis, suggesting that most autoantibodies are associated with neither antitumor immunity nor protumor effects. Examples of this association are as follows: high levels of autoantibodies to MUC1 in patient sera are associated with favorable prognosis in a relatively small cohort of breast cancer patients.^8^ Nevertheless, a study involving a large patient cohort and healthy controls demonstrated that the levels of anti‐MUC1 autoantibodies in patient sera are similar to those of healthy controls, suggesting that autoimmunity to MUC1 in breast cancer may not be enhanced.^9^ Moreover, the high levels of serum anti‐TP53 autoantibodies are shown to be associated with poor outcomes in breast cancer patients.^10^ This finding suggests that autoimmunity to TP53 is pro‐tumorigenic or that a high level of anti‐TP53 autoantibodies simply reflects a high tumor burden and/or mutated TP53 gene, representing increased aggressiveness of the tumor, thereby anti‐TP53 autoantibodies may not provide prognostic information in addition to the preexisting clinicopathological factors.

Given the production of autoantibody to a TAA is associated with enhanced antitumor immunity, it is speculated that the level of autoantibodies in cancer patients is lower than that in healthy individuals because healthy individuals may be protected against cancer by the enhanced antitumor immunity. Thus, to identify an autoantibody associated with enhanced antitumor immunity, a highly sensitive quantitative assay that can determine low levels of autoantibody needs to be established. Using a highly sensitive ELISA assay, we found that the serum concentration of autoantibody to HER2 (HER2‐AAb) in patients with early stage breast cancer is significantly lower (*p* = 1.8 × 10^–7^) than that in healthy subjects.^11^ We chose to investigate HER2‐AAb as the autoantibody specifically because it is detected in both healthy individuals and those with breast cancer.^12^, ^13^ Further, inferring from the success of trastuzumab‐based HER2‐targeted therapy, we hypothesized that HER2‐AAb might have antitumor effects. In line with this hypothesis, we found that breast cancer patients with a high serum concentration of HER2‐AAb had a significantly lower recurrence rate after surgery than those with a low serum concentration of HER2‐AAb (hazard ratio =0.13; log‐rank *p* = 0.015). These findings suggest that a high level of HER2‐AAb is associated with antitumor immunity against breast cancer. Thus, we aimed to determine whether and how antitumor immunity was enhanced in breast cancer patients whose serum concentration of HER2‐AAb was high.

## 2 MATERIALS AND METHODS

### 2.1 Patients

**FIGURE 1:**
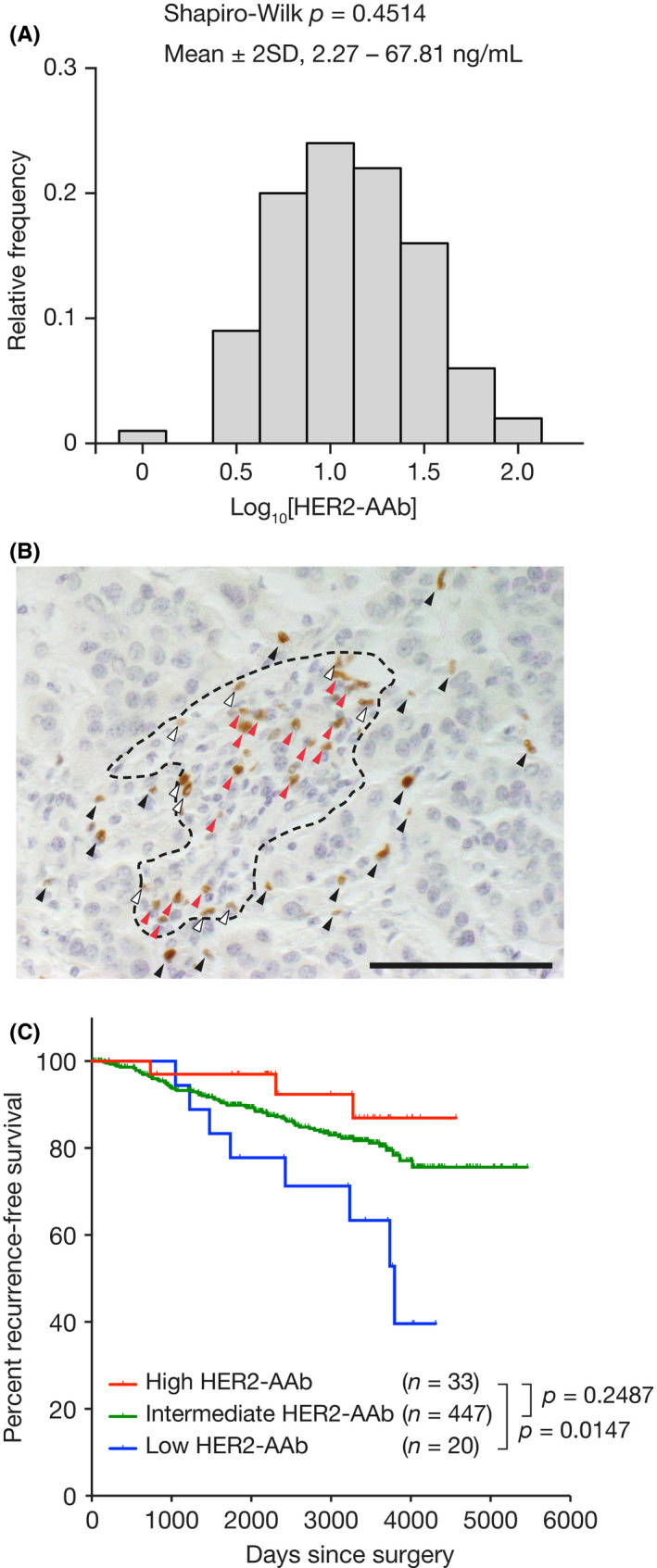
(A) Distribution of the log‐transformed serum anti‐HER2 autoantibody (HER2‐AAb) concentrations of 100 healthy individuals. Shapiro‐Wilk test was performed to demonstrate the normality of the distribution. (B) An example of IC counting. FOXP3‐positive ICs infiltrating the IT (black arrowheads), AS (white arrowheads), and distant stromal area (red arrowheads) are indicated. The area surrounded by a broken line denotes the tumor stroma. Original magnification, ×40. IC, immune cell; IT, intratumor area; AS, adjacent stromal area. (C) Kaplan–Meier curves of patients with high HER2‐AAb concentration (red line, HER2‐AAb >67.81 ng/ml), intermediate HER2‐AAb concentration (green line, 2.27 ng/ml ≤HER2‐AAb ≤67.81 ng/ml), and low HER2‐AAb concentration (blue line, HER2‐AAb <2.27 ng/ml). Percentages of recurrence‐free survival are shown. *p*, log‐rank test. Scale bar, 100 µm

The distribution of the log‐transformed serum HER2‐AAb concentrations of 100 healthy women (median age, 60 years [range, 35–76]) recruited at a breast screening center reported previously,^11^ demonstrated a normal distribution (Figure 1A). The range between mean −2SD and mean +2SD values of the healthy cohort was calculated to be between 2.27 ng/ml and 67.81 ng/ml. Among the 500 consecutive patients with operable invasive breast cancer whose presurgical serum HER2‐AAb concentrations had been measured,^11^ we selected those whose HER2‐AAb values were below 2.27 ng/ml (low HER2‐AAb group, *n* = 20) or above 67.81 ng/ml (high HER2‐AAb group, *n* = 33).

### 2.2 Immunohistochemistry

Tumor formalin‐fixed paraffin‐embedded (FFPE) samples (*n* = 31, high HER2‐AAb group; *n* = 18, low HER2‐AAb group) and regional lymph node FFPE samples (*n* = 29, high HER2‐AAb group; *n* = 18, low HER2‐AAb group) prepared from the patients’ surgical specimens were subjected to immunohistochemistry. Several cases in the cohort were not used solely because the corresponding FFPE samples were missing. The FFPE blocks were sliced at a thickness of 4 μm, and the thin sections were de‐paraffinized with Hemo‐De (Falma) and ethanol followed by rehydration with 80% ethanol and tap water. For CD4 and CXCR5 double staining and for CXCL13 and PD‐L1 staining, the thin sections were fixed again with 10% buffered formaldehyde for 2 min and 10 mM glycine‐PBS for 10 min. Antigen retrieval was performed with Target Retrieval Solution pH 6 (Dako) at 98°C for 20 min for CD20 and IGKC, with Target Retrieval Solution pH 9 (Dako) at 98°C for 20 min for HLA‐A/B/C, with Target Retrieval Solution pH 9 at 98°C for 40 min for CD8 and FOXP3, with Target Retrieval Solution pH 9 at 121°C for 10 min for CXCL13 and CD4 plus CXCR5 double staining, and with Universal HIER Antigen Retrieval Reagent pH 6 (Abcam) at 125°C for 1 min for PD‐L1. Inactivation of endogenous peroxidase was performed with 2% H_2_O_2_/methanol for 10 min, and blocking was performed with an Avidin/Biotin Blocking Kit (Vector Laboratories) and/or Block ACE (KAC) for 30 min. The following primary antibodies were used: polyclonal anti‐Kappa Light Chain (1:200, Invitrogen), anti‐CD20 (L26, 1:50, Abcam), anti‐HLA Class 1 ABC (EMR8‐5, 1:100, Abcam), anti‐CD8 (C8/144B, 1:100, Dako), anti‐FOXP3 (236A/E7, 1:100, Abcam), anti‐CXCR5 (D6L3C, 1:200, Cell Signaling Technology), anti‐CD4 (4B12, 1:80, Dako), polyclonal anti‐CXCL13 (1:100, Invitrogen), and SP142‐compatible anti‐PD‐L1 (28‐8, 1:400, Abcam).^14^ The specimens were incubated with the primary antibodies overnight at 4°C. Then, the specimens were incubated at room temperature for 30 min or 1 h with the corresponding secondary antibodies, including biotin SP‐conjugated donkey anti‐rabbit antibody (1:100, Jackson ImmunoResearch) for IGKC; biotin SP‐conjugated donkey anti‐mouse antibody (1:200, Jackson ImmunoResearch) for CD20; Histofine Simple Stain MAX PO (M) (Nichirei Bioscience) for HLA‐A/B/C, CD8, and FOXP3; and Histofine Simple Stain MAX PO (R) (Nichirei Bioscience) for CXCL13. Detection was carried out with DAB substrate. For IGKC and CD20, signal amplification with Vectastain ABC Kit Peroxidase (Vector) was carried out before detection. For PD‐L1, rabbit‐specific immunohistochemistry (IHC) polymer detection kit HRP/DAB (Abcam) was used in accordance with the manufacturer's instruction. For double staining with CD4 and CXCR5, DoubleStain IHC Kit: M&R on human tissue (Abcam) was used in accordance with the manufacturer's instruction. Hematoxylin was used for counterstaining. HER2 staining was performed with a HercepTest II Kit (Dako) in accordance with the manufacturer's instructions. Conventional protocol was followed for dehydration, clearing, and cover‐slipping.

### 2.3 Histological evaluations

Tumor‐infiltrating immune cells (ICs) were separately counted in three regions: intratumoral (IT) ICs, defined as ICs present in tumor parenchyma, adjacent stromal (AS) ICs, defined as ICs present within a tumor cell (TC) diameter away from tumor parenchyma, and distant stromal ICs, defined as ICs present in a tumor stroma but outside of an AS compartment. A representative microphotograph of a breast tumor in which ICs were present in the IT, AS, and distal stromal area is shown in Figure 1B.

Each cell count was a sum of the counts in five 40× fields selected randomly. Total cell count was defined as a sum of IT, AS, and distant stromal ICs. Follicular CD4‐positive cells were defined as CD4‐positive cells in CXCR5‐positive cell clusters/follicles in the axillary lymph nodes and demonstrated as a sum of five 40× fields selected randomly. Protein expression in TCs was evaluated as a percentage of TCs positive for the protein of interest except for HER2. HER2 expression was estimated in accordance with the recommendation by the American Society of Clinical Oncology and the College of American Pathologists.^15^ For cell counting, a researcher (YaS) randomly selected five 40× fields for each specimen and manually counted all ICs present in each field using the BX51 light microscope (Olympus). Another researcher (MS) verified whether the cells were correctly counted without knowledge of sample identification. To evaluate protein expression including that of HER2, YaS first evaluated the degree of protein expression, and MS verified the evaluation thereafter without knowledge of sample identification. Further, YaS investigated the proportion of tumor‐infiltrating lymphocytes (TILs) according to the recommendation by the International TILs Working Group,^16^ and a pathologist (Dr. Keiichirou Homma) in our institution reviewed the evaluation.

### 2.4 Repertoire analysis for B‐cell receptors (BCRs)

Among patients whose frozen tumor samples obtained by surgical removal were available, the top 10 patients in the high HER2‐AAb group and top five patients in the low HER2‐AAb group—those with the highest numbers of CD20‐positive ICs infiltrating the tumors were selected. The frozen samples were immersed in RNAlater‐ICE (Invitrogen) and stored at –80°C until use. RNA extraction, unbiased amplification of BCR genes, next‐generation sequencing, and data analysis were performed by Repertoire Genesis Inc. as previously described.^17^, ^18^

### 2.5 HER2 fluorescent in situ hybridization

HER2 gene amplification of TCs from surgically excised tumors in all selected cases was examined by SRL, Inc. Briefly, FFPE samples were sectioned at a thickness of 4 μm and stained with a PathVysion HER2 DNA Probe Kit (Abbott) in accordance with the manufacturer's instructions. Fluorescence was observed using Duet‐3 (BioView). HER2 amplification was evaluated in accordance with the recommendation by the American Society of Clinical Oncology and the College of American Pathologists.^15^

### 2.6 HER2 mutation analysis

We selected patients whose frozen tumor samples were available for further analysis (23 patients in the high HER2‐AAb group and 15 patients in the low HER2‐AAb group). The frozen samples were subjected to genomic DNA extraction using the DNeasy Blood & Tissue Kit (Qiagen). One sample from the high HER2‐AAb group and one sample from the low HER2‐AAb group were not used for further analysis because of insufficient DNA content. The library was prepared using xGen custom probes for all exons of the *HER2*/*ERBB2* gene (IDT), followed by next‐generation sequencing using MiSeq (Illumina). The obtained sequences were mapped to the *hg19* human reference sequence to detect mutations. Among the detected mutations, synonymous mutations, single‐nucleotide polymorphisms described in the dbSNP Database, those with less than 10 mutant allele coverages, germline mutations with around 50% or 100% mutant allele frequencies, and mutations with fewer than 8% mutant allele frequencies were excluded.

### 2.7 Statistics

Statistical tests used are specified in the figures, figure legends, and table footnotes. Statistical analysis was performed using GraphPad Prism 6.0 (GraphPad Software, Inc.) and JMP Pro 11 (SAS Institute Inc.). A *p* value of <0.05 was considered significant. All tests were described as two‐tailed.

### 2.8 Ethics approval and consent to participate

All procedures performed in this study were in accordance with the ethical standards of the institutional ethical committee (Osaka University Hospital Clinical Research Review Committee, reference #14046‐3) and with the 1964 Helsinki Declaration and its later amendments or comparable ethical standards. Written informed consent was obtained from all individual participants included in the study.

### 2.9 Consent for publication

Consent for the publication of research data was included in the consent forms and approved by the institutional review board of Osaka University Hospital. Images from tissue specimens of patients are entirely unidentifiable.

### 2.10 Data availability statement

All data generated or analyzed during this study are included in this article and its additional files.

## 3 RESULTS

### 3.1 Patient selection and prognosis of patients selected in the present study

We aimed to compare the immunological microenvironment of breast tumors in patients with high levels of serum HER2‐AAb to that of breast tumors in patients with low levels of HER2‐AAb. The log‐transformed serum HER2‐AAb concentrations of 100 healthy individuals reported previously^11^ demonstrated normal distribution (Figure 1A). Thus, 500 patients with invasive breast cancer, previously reported in a study,^11^ were divided into three groups (high, intermediate, and low HER2‐AAb groups) depending on two cutoffs: log‐transformed HER2‐AAb concentrations at mean +2SD and mean –2SD of the healthy control. Kaplan–Meier curves of recurrence‐free survival showed that the high HER2‐AAb group had significantly longer recurrence‐free survival than the low HER2‐AAb group, while the intermediate HER2‐AAb group had an outcome in between (Figure 1C). Accordingly, the high HER2‐AAb group (*n* = 33) and the low HER2‐AAb group (*n* = 20) were selected for further analyses. The baseline patient characteristics of the two groups were similar in terms of menopausal status, histological type, tumor size, lymph node metastasis, histological grade, hormone receptor status, HER2 status, and adjuvant therapy (Table 1).

**TABLE 1:** Clinicopathological characteristics of invasive breast cancer patients and their HER2‐AAb status

	High HER2‐AAb (*n* = 33)		Low HER2‐AAb (*n* = 20)		*p*
	No.	%	No.	%	
Menopausal status					0.20^a^
Premenopausal	6	18	7	35	
Postmenopausal	27	82	13	65	
Histological type					0.19^b^
IDC	25	76	18	90	
ILC	5	15	0	0	
Others	3	9	2	10	
T status					0.75^a^
1	26	79	15	75	
2 and 3	7	21	5	25	
Lymph node metastasis					1.00^a^
Negative	24	73	15	75	
Positive	9	27	5	25	
Histological grade					0.64^a^
1 and 2	29	88	19	95	
3	4	12	1	5	
HR status					0.72^a^
Negative	5	15	4	20	
Positive	28	85	16	80	
HER2 status					1.00^a^
Negative	26	79	16	80	
Positive	7	21	4	20	
Adjuvant therapy					0.26^b^
No treatment	0	0	2	10	
Endocrine therapy^c^	17	52	11	55	
Chemotherapy^c^	3	9	1	5	
Chemo‐endocrine^c^	8	24	4	20	
Trastuzumab	5	15	1	5	
Unknown	0	0	1	5	

Abbreviations: HER2‐AAb, anti‐HER2 autoantibody; HR, hormone receptors; IDC, invasive ductal carcinoma; ILC, invasive lobular carcinoma.

^a^ Fisher's exact test.

^b^ *χ* ^2^ test.

^c^ Patients treated with trastuzumab are not included.

### 3.2 Association of HER2‐AAb with tumor‐infiltrating ICs

**FIGURE 2:**
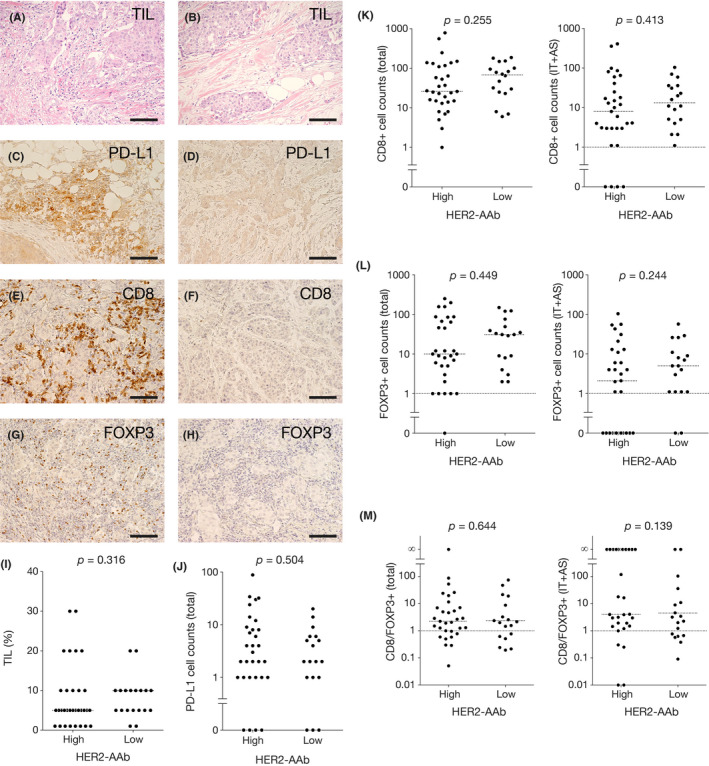
Tumor‐infiltrating lymphocytes (TILs), PD‐L1‐positive ICs, CD8‐positive ICs, FOXP3‐positive ICs, and CD8‐positive‐to‐FOXP3‐positive ratios in the high and low HER2‐AAb groups. (A and B) Representative photographs of hematoxylin‐eosin staining of the tumor with high TIL content (A) and of the tumor with low TIL content (B). (C–H) Representative photographs of immunohistochemical (IHC) staining for PD‐L1 (C and D), CD8 (E and F), and FOXP3 (G and H) of the tumors with large cell counts (C, E and G) and the tumors with small cell counts (D, F and H). Original magnification, ×20. (I) Percent TIL content in the high HER2‐AAb group and the low HER2‐AAb group. (J–L) Counts of ICs positive for PD‐L1 (J), CD8 (K), and FOXP3 (L) in the high HER2‐AAb group and the low HER2‐AAb group. Total denotes the cells in IT, AS, and the distant stroma, while IT+AS denotes the cells in IT and AS. (M) CD8‐positive‐to‐FOXP3‐positive ratios in the high HER2‐AAb group and the low HER2‐AAb group. Medians are shown by horizontal dotted lines. *p*, Mann–Whitney *U* test. Scale bar, 100 µm

Tumor‐infiltrating lymphocytes (TILs) play a central role in antitumor immunity. Thus, we aimed to investigate the association between HER2‐AAb and the extent of TILs and to clarify the difference in various IC types infiltrating in the tumors between the high and low HER2‐AAb groups. The extent of TILs in the high HER2‐AAb group was similar to that in the low HER2‐AAb group (Figure 2A, B and I). Immunosuppressive PD‐L1‐positive immune cell counts (ICCs) were also similar between the two groups (Figure 2C, D and J). CD8‐positive cytotoxic T cells and FOXP3‐positive regulatory T cells are key players in antitumor immunity that positively and negatively correlate with the prognosis of breast cancer patients, respectively.^19^ The total (i.e., IT, AS, and distant stromal) CD8‐positive ICCs, IT plus AS (IT+AS) CD8‐positive ICCs, total FOXP3‐positive ICCs, and IT+AS FOXP3‐positive ICCs were similarly distributed in both the high and low HER2‐AAb groups (Figure 2E–H, K and L). The ratio of CD8‐positive ICCs to FOXP3‐positive ICCs, another indicator of cellular antitumor immunity, was also similar between the two groups, although a trend of high ratio of IT+AS CD8‐positive ICCs to IT+AS FOXP3‐positive ICCs was found in the high HER2‐AAb group as compared to the low HER2‐AAb group (Figure 2M).

**FIGURE 3:**
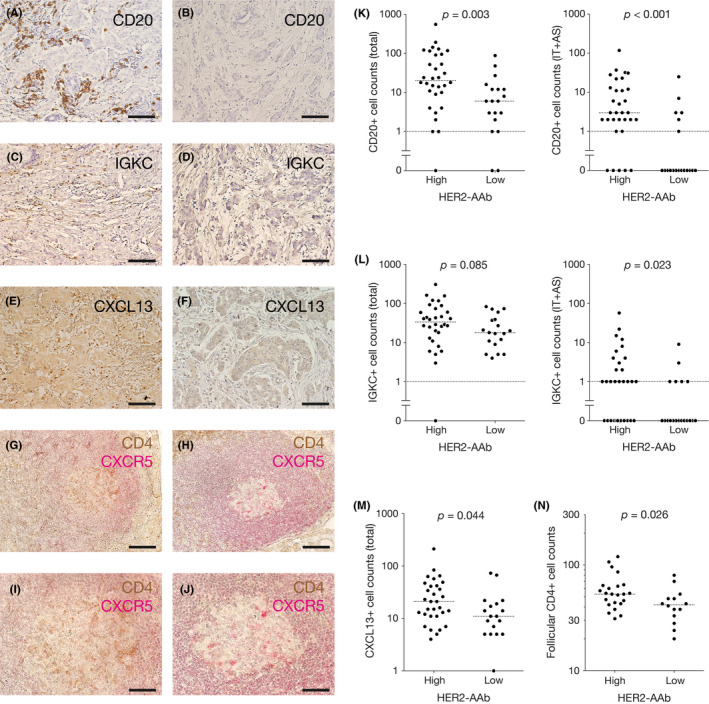
Tumor‐infiltrating CD20‐positive ICs, IGKC‐positive ICs, and CXCL13‐positive ICs, and follicular CD4‐positive ICs in the regional axillary lymph nodes in the high HER2‐AAb group and the low HER2‐AAb group. (A–F) Representative photographs of IHC staining for CD20 (A and B), IGKC (C and D), and CXCL13 (E and F) of the tumors with large cell counts (A, C and E) and the tumors with small cell counts (B, D and F). (G–J) Representative photographs of IHC staining for CD4 (brown) and CXCR5 (red) of the regional axillary lymph nodes with large cell counts (G and I) and with small cell counts (H and J). Images (I) and (J) are the high power fields of the images (G) and (H), respectively. Original magnification, ×20 (A–H) and ×40 (I and J). (K–M) Counts of ICs positive for CD20 (K), IGKC (L), and CXCL13 (M) in the high HER2‐AAb group and the low HER2‐AAb group. (N) Counts of CD4‐positive ICs in CXCR5‐positive B‐cell follicles (follicular CD4^+^) of the regional axillary lymph nodes in the high HER2‐AAb group and the low HER2‐AAb group. Medians are shown by horizontal dotted lines. *p*, Mann–Whitney *U* test. Scale bar, 100 µm (A–H) and 50 µm (I and J)

B cells and plasma cells play a pivotal role in antibody production. Total and IT+AS ICCs positive for a pan‐B cell marker CD20 in the high HER2‐AAb group were significantly greater than those in the low HER2‐AAb group (Figure 3A, B and K). The level of IT+AS, but not total, ICCs positive for IGKC, a plasma cell marker, in the high HER2‐AAb group was also significantly greater than those in the low HER2‐AAb group (Figure 3C, D and L), suggesting that tumor‐infiltrating B cells and plasma cells were involved in the production of HER2‐AAb. CXCL13 is a major chemoattractant for B cells.^20^ The total amount of CXCL13‐producing ICCs was significantly greater in the high HER2‐AAb group than in the low HER2‐AAb group (Figure 3E, F and M), suggesting that the greater infiltration of B cells in the high HER2‐AAb group was due to the infiltration of CXCL13‐producing ICs.

### 3.3 Association of HER2‐AAb with follicular CD4‐positive ICs in the secondary lymphoid organs (SLOs)

Follicular helper T (Tfh) cells enable the maturation of B cells in the lymphoid follicles of SLOs, so they are essential for humoral immunity.^21^, ^22^ Classical Tfh cells are positive for CD4, CXCR5, ICOS, PD‐1, and BCL6. Identification of Tfh cells by flow cytometry is relatively easy because several markers can be simultaneously stained, and the expression intensities of the markers can be estimated. However, the information on the localization of Tfh cells in lymphoid tissues is lost. Identification of Tfh cells by IHC using FFPE tissue samples, which are usually the only accessible samples obtained from cancer patients, is challenging because the multiple‐staining technique is relatively difficult and double‐positive staining is often not apparent. The advantage of IHC is that the localization of cells of interest is clearly evaluable. We considered the major proportion of an IC population expressing CD4 in a B‐cell follicle, identified by a CXCR5‐positive cell cluster, to consist of Tfh cells, though a minor population such as follicular regulatory T cells could not be excluded. The sentinel lymph nodes of the axilla are the SLOs of the mammary gland that needs to be removed during mastectomy or lumpectomy in breast cancer surgery. Thus, we examined the number of CD4‐positive lymphocytes in CXCR5‐positive B‐cell follicles of the excised sentinel lymph nodes and/or axillary lymph nodes. We found that CD4‐positive lymphocytes were present at a significantly greater level in the B‐cell follicles of the regional lymph nodes of the high HER2‐AAb group than of the low HER2‐AAb group (Figure 3G–J, N).

### 3.4 Repertoire analysis for BCRs in TILs

**FIGURE 4:**
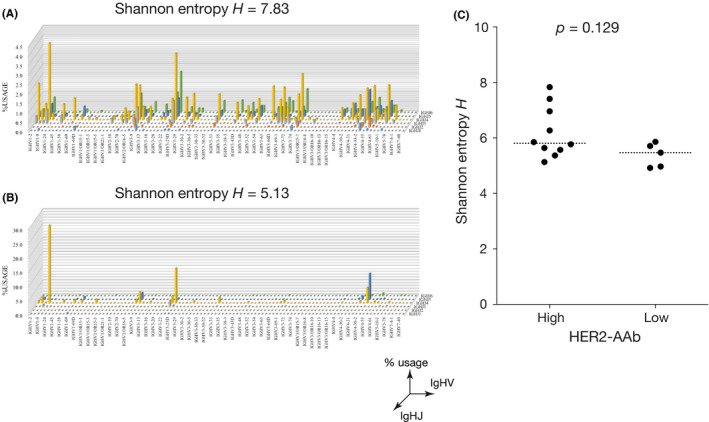
Repertoire analysis for B‐cell receptors (BCRs) in TILs. (A and B) Examples of IgHVJ repertoire 3D histograms (% usage). X‐axis and Y‐axis denote IgHV repertoire and IgHJ repertoire, respectively. The diversity of BCRs was estimated by Shannon entropy *H*. (C) Shannon entropies of BCR repertoires of tumor‐infiltrating ICs. Medians are shown by horizontal dotted lines

The diversity of BCRs in TILs reflects the specificity of the humoral immune responses in the tumor microenvironment.^23^ To determine whether tumor‐infiltrating B cells of the high HER2‐AAb group responded to specific TAAs including HER2, we examined the mRNA sequence of the CDR3 region of the *IgHV* and *IgHJ* genes by the unbiased amplification of BCR genes followed by next‐generation sequencing.^17^, ^18^ Although Shannon entropy scores, *H*, indicating the diversity of BCRs, were not significantly different between the high HER2‐AAb group and low HER2‐AAb group, the IgHVJ distributions were skewed in most of the cases of both groups (Figure 4; Figure S1), suggesting that tumor‐infiltrating B cells were involved in specific immune responses regardless of the HER2‐AAb status.

### 3.5 Tumor factors associated with HER2‐AAb

**FIGURE 5:**
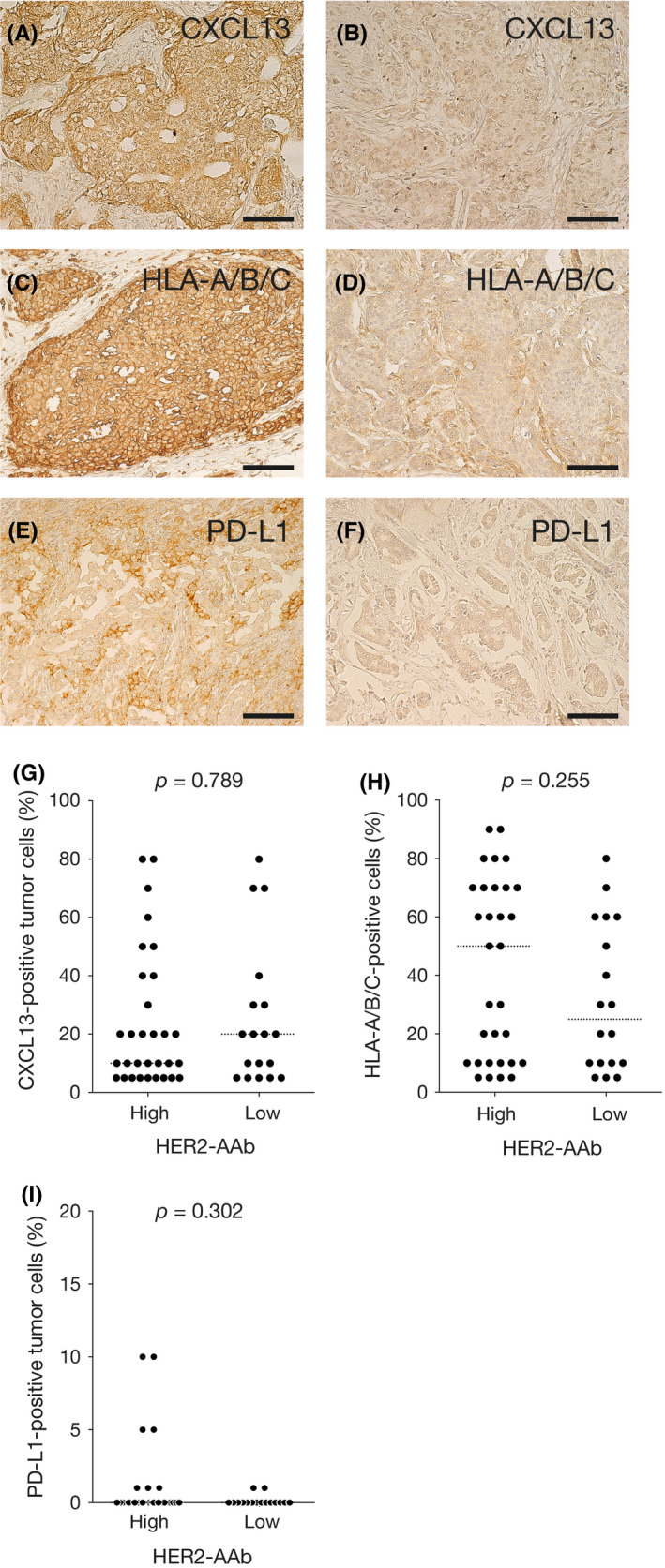
Tumor factors associated with HER2‐AAb. (A–F) Representative photographs of IHC staining for CXCL13 (A and B), HLA‐A/B/C (C and D), and PD‐L1 (E and F). Highly expressing tumor cells (A, C and E) and minimally expressing tumor cells (B, D and F) are shown. Original magnification, ×20. (G–I) Percent tumor cells positive for CXCL13 (G), HLA‐A/B/C (H), and PD‐L1 (I) in the high HER2‐AAb group and low HER2‐AAb group. Medians are shown by horizontal dotted lines. *p*, Mann–Whitney *U* test. Scale bar, 100 µm

Tumors may shape their microenvironments by expressing a number of immune‐related cell surface molecules and by secreting cytokines and chemokines that recruit or repel specific types of stromal cells or ICs. A B‐cell‐specific chemokine CXCL13 was expressed in tumors of the high and low HER2‐AAb groups to the same extent (Figure 5A, B and G). HLA‐A/B/C is the human major histocompatibility complex molecule essential for the recognition of TAAs by ICs. Loss of HLA‐A/B/C expression on TC surface leads to escape from immunosurveillance. The extent of HLA‐A/B/C expression on the TCs was not significantly different between the high and low HER2‐AAb groups (Figure 5C, D and H). PD‐L1 expression on the TCs was generally weak or absent, which is consistent with the previous report,^24^ and the difference in the expression levels of PD‐L1 between the two groups was not observed (Figure 5E, F and I).

**FIGURE 6:**
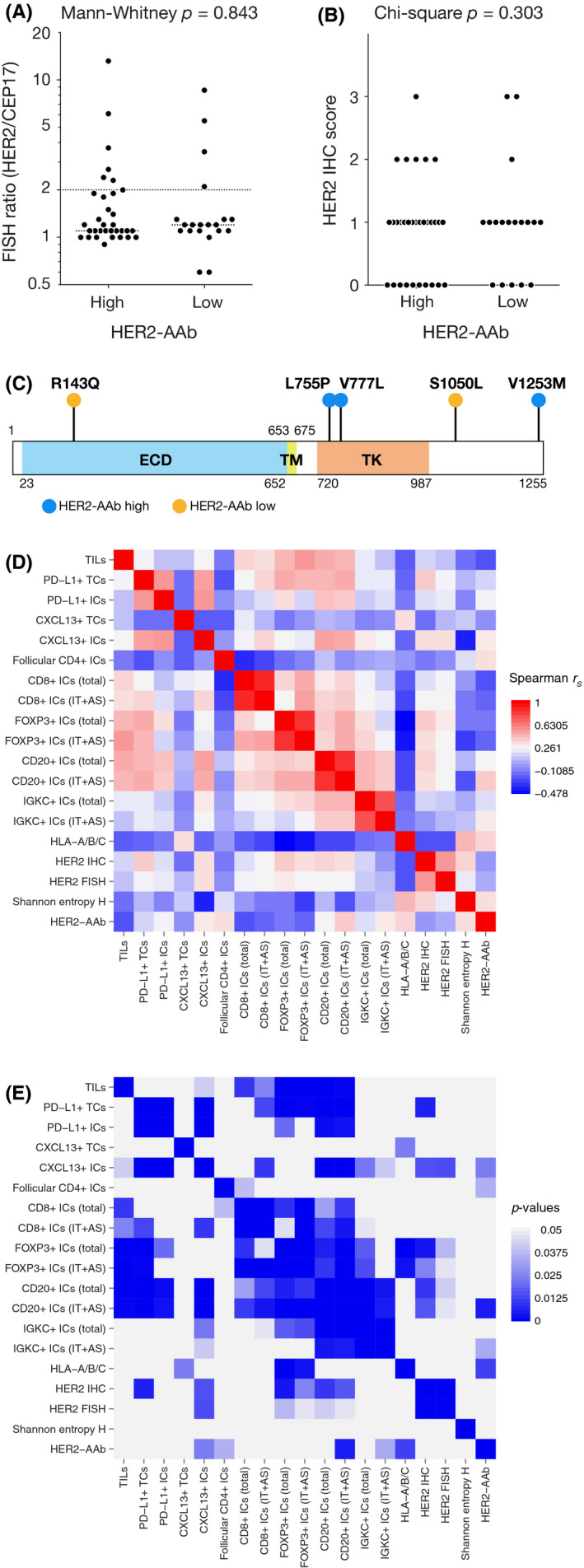
(A–C) HER2 status in the high HER2‐AAb group and low HER2‐AAb group. (A) *HER2* gene amplification estimated by fluorescence in situ hybridization (FISH). HER2 copy number was standardized with CEP17 and is demonstrated by the HER2‐to‐CEP17 FISH ratio. (B) HER2 expression estimated by the standard IHC scoring. (C) Missense somatic mutations in the *HER2* gene. In 38 analyzed tumor samples, five tumors had missense mutations in the *HER2* gene. ECD, extracellular domain; TM, transmembrane domain; TK, tyrosine kinase domain. (D and E) Association between two factors among all factors evaluated in the present study. Spearman's rank correlation coefficients *r_S_* (D) and the corresponding *p* values (E) are shown by heat maps

We explored the abnormality of the *HER2* gene to determine whether it was associated with HER2‐AAb. The amplification of the *HER2*/*ERBB2* gene and the expression of HER2 protein, estimated by fluorescent in situ hybridization and immunohistochemistry, respectively, were not different between the high and low HER2‐AAb groups (Figure 6A and B). Missense somatic mutations in the *HER2* gene were observed in 3 out of 23 cases in the high HER2‐AAb group and in 2 out of 15 cases in the low HER2‐AAb group (Figure 6C).

### 3.6 Association between two factors among IC factors, TC factors, and HER2‐AAb

Finally, we evaluated the association between the two factors in light of the various factors estimated in the present study (Figure 6D and E). Spearman's rank correlations of the two factors confirmed that tumor‐infiltrating CXCL13‐positive ICs, CD20‐positive ICs, and IGKC‐positive ICs, and follicular CD4‐positive ICs in the regional lymph nodes were positively associated with HER2‐AAb. In addition, HLA‐A/B/C expressed in TCs was also positively associated with HER2‐AAb. Tumor‐infiltrating CD8‐positive ICs, FOXP3‐positive ICs, CD20‐positive ICs, and IGKC‐positive ICs generally had positive correlations with each other. Of note, tumor‐infiltrating CXCL13‐positive ICs were positively correlated with CD20‐positive ICs and IGKC‐positive ICs, suggesting that the tumor infiltration of B cells and plasma cells was due to CXCL13 production by TILs. HER2 expression and amplification were positively associated with tumor‐infiltrating CXCL13‐positive ICs and CD20‐positive ICs as well as FOXP3‐positive ICs.

## 4 DISCUSSION

We found that the high concentration of serum HER2‐AAb was associated with the accumulation of B cells, plasma cells, and CXCL13‐positive ICs in breast tumors and of follicular CD4‐positive ICs, which presumably represented Tfh cells, in the axillary lymph nodes. The accumulation of these IC types in the high HER2‐AAb group was unlikely to be a coincidence because those cells are functionally relevant to each other in the context of the enhancement of humoral immunity; Tfh cells play a central role in enhancing humoral immunity by interacting with the B cells in the germinal center of B‐cell follicles in the SLOs.^21^ The stimulated B cells in SLOs differentiate mainly into two distinct cell types: antibody‐producing plasma cells and long‐term memory B cells preparing for the second attack by foreign bodies. Therefore, an increase in CD4‐positive cells in the B‐cell follicles of the axillary lymph nodes in the high HER2‐AAb group strongly supports that humoral immunity was enhanced in the high HER2‐AAb group. CXCL13, a chemokine that strongly attracts B cells,^20^ is produced by Tfh cells as well as follicular dendritic cells in breast tumor tissues and plays a pivotal role in enhancing antitumor humoral immunity.^25^, ^26^ In this study, an increase in the CXCL13‐positive ICs in the high HER2‐AAb group indicates that the CXCL13‐positive ICs in the tumor stroma and the follicular CD4‐positive ICs found in the SLOs both stimulated the maturation of B cells and helped them to migrate to the breast tumor stroma. Interestingly, CXCL13 expression in TCs was not associated with HER2‐AAb or ICCs, suggesting that B‐cell stimulation with CXCL13 needed the engagement of MHC class II on B cells and T‐cell receptors on CXCL13‐secreting T cells in addition to co‐stimulatory molecule(s) such as CD40/CD40L, and ICOS/ICOSL.^27^ The interaction between B and T cells can occur in the tumor stroma, especially in tumor stromal lymphoid tissues, namely tertiary lymphoid structures^28^; notably, B cells in the tumor stroma can cross‐present tumor antigens to cytotoxic T cells and stimulate them to attack TCs.^29^ Consistent with this evidence is our result that CD20‐positive ICCs are positively associated with CD8‐positive ICCs in the present cohort. Altogether, our results indicate that in the high HER2‐AAb group, antitumor humoral immune responses were systematically enhanced, leading to the production of HER2‐AAb.

The association between favorable prognosis and a high level of serum HER2‐AAb has been demonstrated in a previous study,^11^ and it was confirmed in the selected cohort examined in the present study. To date, B‐cell or plasma‐cell signatures in breast tumors estimated by microarray or next‐generation sequencing techniques have been found to be linked to favorable outcomes in patients with breast cancer.^30^, ^31^ Consistently, the extent of tumor‐infiltrating B cells and plasma cells estimated by IHC is correlated with favorable outcomes for this disease,^32^, ^33^ indicating that humoral immunity is important for antitumor immunity against breast cancer as compared to other cancer types, in which cellular immunity generally plays a pivotal role. Tfh cells residing in the tumor stroma are also a distinct IC type associated with favorable breast cancer prognosis,^25^ owing to their function of facilitating B‐cell differentiation. In fact, an increased number of CD20‐positive B cells in sentinel lymph nodes, stimulated presumably with Tfh cells, is associated with a favorable prognosis.^34^ The prognostic impact of CXCL13 is still controversial, probably because CXCL13 plays a distinct role depending upon the cancer type. However, for early breast cancer, high CXCL13 content in a tumor is independently associated with favorable outcomes,^35^ supporting the importance of CXCL13 for humoral immune responses in anti‐breast cancer immunity. Such evidence indicates that humoral immunity plays a substantial role in the breast cancer microenvironment. Thus, findings from this study may provide a rationale for the association of HER2‐AAb with the prognosis of breast cancer patients.

Notably, the rationale behind the immunogenicity of HER2 is still unclear. In this present study, we observed an increased number of patients with invasive lobular carcinoma in the high HER2‐AAb group, albeit not statistically significant. Missense mutations can be found more frequently in the *HER2* gene of invasive lobular carcinoma than in the *HER2* gene of invasive ductal carcinoma.^36^ Thus, we hypothesized that the frequency of missense mutations, which may cause the generation of neoepitopes, would be higher in the high HER2‐AAb group than in the low HER2‐AAb group. However, the frequencies of the missense mutations in the high and low HER2‐AAb groups were similar in the present cohort, suggesting that the immunogenicity of HER2 cannot be not attributed to the generation of neoepitopes. This finding is in line with the studies on anti‐HER2 vaccines that identified two potential epitopes, E75 and GP2, derived from the wild‐type HER2 polypeptide. Both inherent epitopes have been reported to induce immune responses through cytotoxic T cells.^37^, ^38^ If so, it can be postulated that the immunogenicity of HER2 may depend on the abundant HER2 protein in the tumor microenvironment. Although we could not demonstrate the correlation between HER2‐AAb and HER2 expression, we observed significant associations of tumor‐infiltrating B cells and CXCL13‐positive immune cells with HER2 expression (Figure 6), suggesting that a certain concentration of HER2 induced a humoral immune response which may have led to the production of HER2‐AAb. Alternatively, the sugar chains in HER2 glycoprotein may be immunogenic, even though sugar chains in an endogenous protein are generally less immunogenic than those in a bacterial protein. Further, the immunogenicity of a specific self‐antigen may be determined by multiple factors including the tumor characteristics, immunologic states of the host such as MHC variability, and physical states of the host as well as the structure and expression of antigens. For example, an intriguing report by Pandey et al. shows that the generation of anti‐HER2 autoantibody may be determined not by HER2 but by other genotypes such as IGKC and FcγR, which is involved in the efficacy of anti‐HER2 therapies.^39^ Therefore, it would be valuable to investigate such factors to elucidate why HER2‐AAb is produced in a specific breast cancer subpopulation. A promising approach for uncovering the rationale behind the immunogenicity of HER2 would be epitope mapping, which we plan in our future study.

For the theoretical understanding of the mechanisms underlying antitumor effects of HER2‐AAb, immunological and non‐immunological mechanisms can be proposed; HER2‐AAb may enhance natural killer cell‐mediated cytotoxicity, which is a major mode of action of trastuzumab. HER2‐AAb may also activate the complement system, resulting in tumor cell lysis.^40^ Further, HER2‐AAb may enhance acquired cellular immunity by augmenting the antigen‐presenting potential of DCs.^41^ As a non‐immunological effect of HER2‐AAb, HER2‐AAb has been shown to inhibit the phosphorylation of HER2 and subsequent downstream signaling, which is similar to the mechanism of action of trastuzumab.^42^ Interestingly, HER2‐AAb levels were determined in 500 patients with invasive breast cancer in a previous study where 28 patients were administered adjuvant trastuzumab in combination with chemotherapy.^11^ In this population, only one patient (HER2‐AAb = 5.8 ng/ml) recurred, suggesting that HER2‐AAb may not affect the function of trastuzumab. Despite the theoretical mechanisms of HER2‐AAb, it is necessary to address the exact role of HER2‐AAb in antitumor immunity.

In conclusion, a high level of HER2‐AAb was associated with the systematic enhancement of humoral immunity in the breast cancer microenvironment. Consistent with the growing evidence that humoral immunity plays a crucial role in antitumor immunity in breast cancer, our findings may provide a rationale underlying the association of a high level of HER2‐AAb with favorable prognosis. Further studies investigating the reason underlying HER2‐AAb induction in a specific population of breast cancer patients are warranted.

## CONFLICT OF INTEREST

The authors declare that they have no conflict of interests.

## Supporting information

Figure S1
