# Supplementary material for: Enhanced humoral immunity in breast cancer patients with high serum concentration of anti‐HER2 autoantibody
Source: Cancer Med. 2021 Jan 27;10(4):1418–30. doi: 10.1002/cam4.3742 (PMC7926031; doi:10.1002/cam4.3742)
Supplement: Supplementary file 1 — Figure S1 [file CAM4-10-1418-s001.pdf]

High HER2-AAb

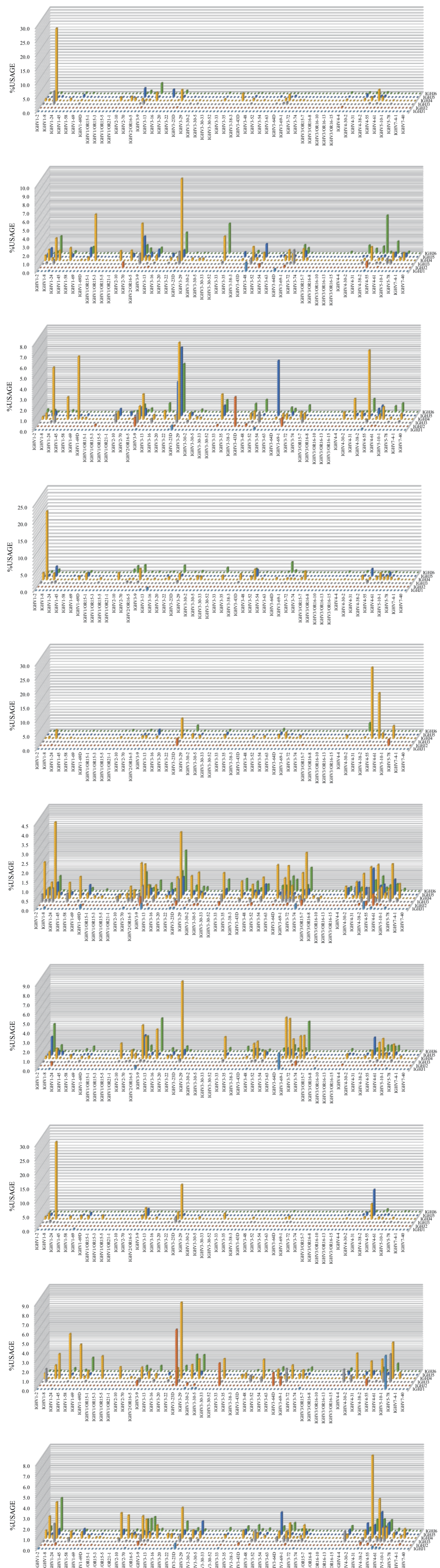

Low HER2-AAb

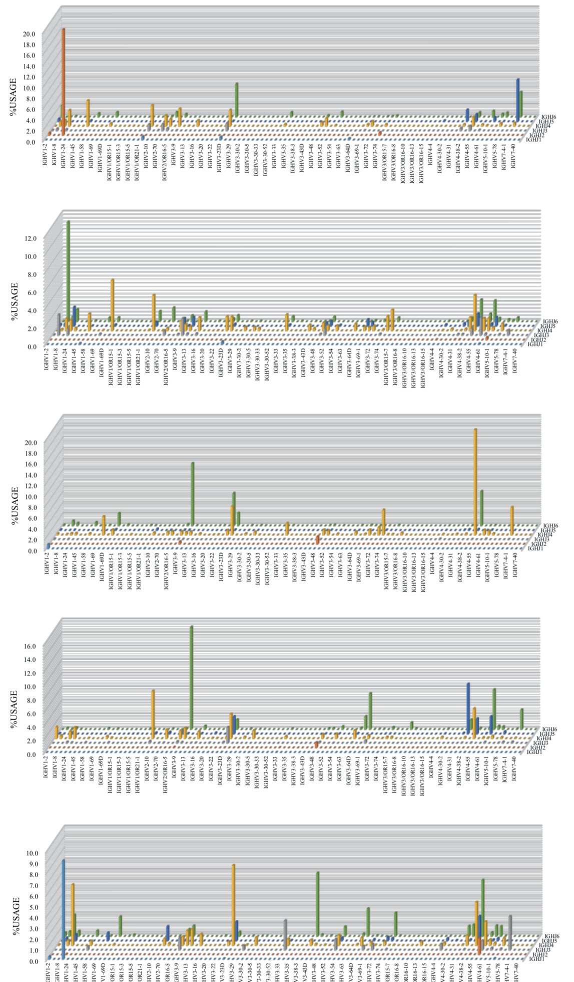

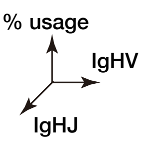

Supplementary Figure 1

Histograms of IgHV (X-axis) and IgHJ (Y-axis) repertoire of all cases examined. Percentages of usage are shown on the Z-axis. The cases in the high HER2-AAb group (*n* = 10, left) and in the low HER2-AAb group (*n* = 5, right) are shown
